# Endoscopic Drainage of an Odontogenic Pterygoid Muscle Abscess

**DOI:** 10.1155/2013/215793

**Published:** 2013-10-10

**Authors:** Rickul Varshney, Faisal Zawawi, Marc A. Tewfik

**Affiliations:** ^1^Department of Otolaryngology—Head & Neck Surgery, McGill University, Royal Victoria Hospital, 687 Avenue Des Pins Ouest, Suite E-4.41, Montreal, QC, Canada H3A 1A1; ^2^Department of Otolaryngology—Head and Neck Surgery, King Abdulaziz University, Jeddah, Saudi Arabia

## Abstract

The infratemporal fossa (ITF) is a potential space bounded by bony structures that can be occupied by both benign and malignant tumors. It is also a potential area of abscess development, most commonly of dental origin. As with any abscess, the treatment of an ITF abscess is surgical drainage. We present a case of an ITF abscess involving the pterygoid muscles following dental extraction in a poorly controlled diabetic patient. The ITF was accessed with an endoscopic transseptal approach through the maxillary sinus to drain the abscess. This case of successful management supports the feasibility of the endoscopic approach in dealing with abscesses of the ITF.

## 1. Introduction 

The infratemporal fossa (ITF) is a potential space bounded by bony structures, namely, the temporal and the sphenoid bones superiorly, the mandible laterally, the pterygoid plates medially, the articular tubercle of the temporal bone and the styloid process posteriorly, and the maxillary sinus anteriorly. The masticator space is one of the deep compartments of the head and neck that contains the muscles of mastication. The medial and lateral pterygoid muscles are shared by both the ITF and the masticator space. The ITF can be occupied by both benign and malignant tumors, which represent less than 1% of head and neck tumors [1]. It is also a potential area for abscess development, most commonly of dental origin [2, 3]. Communications between the ITF, the pterygopalatine fossa (PPF), the parapharyngeal space, the orbit, and the cranial cavity allow contiguous spread of infection between all of these areas.

As with any abscess, the treatment of an ITF abscess is surgical drainage. However, this deep space is not easily accessible, and no consensus exists on the best surgical approach to this region. In fact, surgical access options to the ITF have evolved over time with reports of periauricular, transtemporal, and transmaxillary approaches described by various surgeons [4, 5]. However, morbidities such as facial nerve dysfunction, facial deformities, conductive hearing loss, and dental malocclusion have been reported with these methods [1, 6, 7]. The use of the endoscope to access the ITF via the paranasal sinuses may prevent these morbidities [8]. 

We present a case of ITF abscess involving the pterygoid muscles following dental extraction in a poorly controlled diabetic patient. The ITF was accessed with an endoscopic transseptal approach through the maxillary sinus to drain the abscess. To our knowledge, this is the first report of such an approach for an infectious complication in the ITF. This case of successful management supports the feasibility of the endoscopic approach in dealing with abscesses of the ITF.

## 2. Case Report—Patient A. V

**Figure 1:**
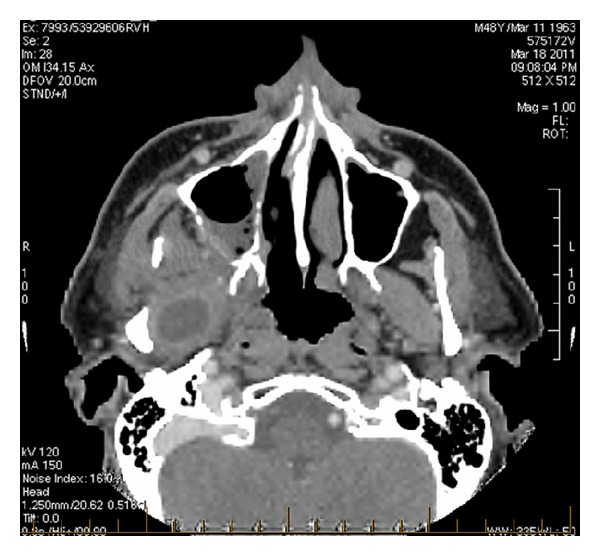
CT axial cut of patient A. V. Right-sided erosion of the posterior wall of the maxillary sinus and a 3.0 cm right lateral pterygoid muscle abscess in the masticator space of the ITF.

 Mr. A. V. is a 48-year-old recently diagnosed diabetic, whose glycemia has been poorly controlled. He presented to our hospital with right-sided facial pain and fever for a few days. He was not able to eat due to significant trismus and throbbing pain. On exam, he had poor dentition and evidence of a right upper tooth infection. Imaging revealed a dental abscess originating from a right molar tooth. He was treated with dental extraction and intravenous antibiotics, then discharged with antibiotics by mouth. One week later, he returned to the hospital with fever and right maxillary facial pain. A CT scan was performed which demonstrated partial right maxillary opacification, erosion of the posterior maxillary wall, and a 3.0 cm right lateral pterygoid muscle abscess in the masticator space (Figure 1). 

**Figure 2:**
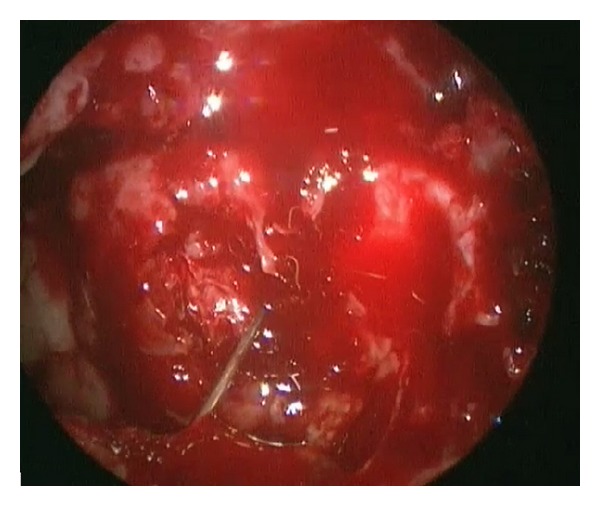
Patient A. V. Needle through posterior wall of maxillary sinus to drain pus.

 The decision was made to perform an endoscopic drainage of the abscess. An endoscopic septoplasty was initially performed via a left Killian incision, and a large rightward bony spur was removed. Then, a right-sided longitudinal mucosal incision was used for transseptal access to drain the abscess. A right middle meatal antrostomy was created, and pus was aspirated from the maxillary sinus. A complete sphenoethmoidectomy was performed, and the inferior half of the middle turbinate was resected to improve access and exposure. A Kerrison punch was used to remove the posterior wall of the maxillary sinus beginning at the sphenopalatine foramen and extending laterally to the ITF using the transseptal access. The periosteum of the pterygopalatine and ITF was seen, and a 25-gauge needle was passed transseptally into the ITF through the periosteum in the direction of the abscess. Two milliliters of pus was aspirated confirming the location of the collection (Figure 2). 

**Figure 3:**
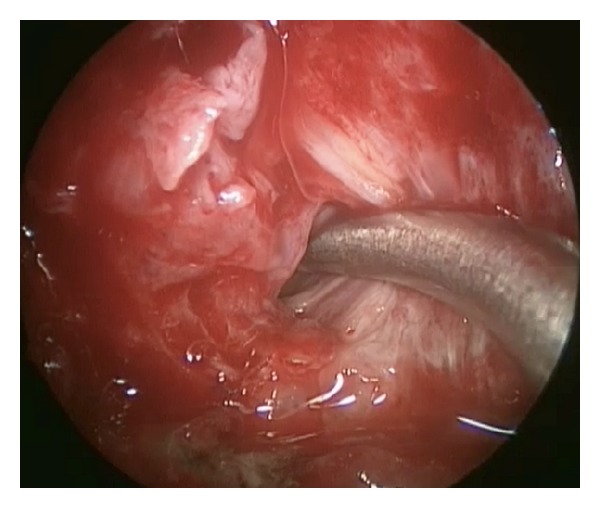
Patient A. V. Sickle knife through posterior wall of maxillary sinus.

 A sickle knife was then used to open the periosteum (Figure 3). The pterygopalatine fossa contents were gently dissected in a lateral direction until the lateral pterygoid muscle was encountered in the ITF. Blunt dissection between the muscle fibers was performed, and an opening to drain the cavity of pus was created. This approach also allowed us to identify and cauterize branches of the internal maxillary artery and adequately irrigate the cavity. The patient had an immediate decrease in pain and facial pressure postoperatively. There were no immediate or delayed complications from the procedure. Postoperatively, the patient was continued on a 6 weeks course of antibiotics for osteomyelitis of the maxillary bone. No recurrence of the abscess has developed until the current 18 month followup.

## 3. Discussion

The masticator space is a deep neck space that harbors the muscles of mastication and shares the medial and lateral pterygoid muscles with the ITF. Its close anatomical relationship to the pterygopalatine fossa, the orbit, and the cranial cavity allows the potential spread of infection between all these areas as well as to deep neck spaces. The patient in this report has an pterygoid muscle abscess from an odontogenic source, which is the most common source of an infection at this site [2, 3]. Yonetsu et al. reported patterns of spread of odontogenic infections and have described the pathway of spread of mandibular infections to the masticator space and then to the greater ITF [9]. Other sources of ITF abscess described include maxillary sinusitis [10], malignant otitis externa [11, 12], and maxillary sinus fracture [12, 13]. Kim et al. reported a case of odontogenic infection spreading from the tooth to the infratemporal fossa progressing further to create an orbital abscess [14].

Trismus is the hallmark sign of ITF abscess [2, 12, 15], secondary to the irritation of the pterygoid muscles located in this space. Trismus was one of the main complaints of our patient, along with fever and facial swelling. Patients can also complain of trigeminal neuralgia type pain as the mandibular branch of cranial nerve V travels in the ITF [15]. 

Many authors have described the Caldwell-Luc procedure [13], a transfacial drainage [2, 14, 15], and an intraoral drainage [12, 15] to access ITF abscesses. Kamath et al. recently published a paper describing a modified Blair incision to drain an ITF abscess in a diabetic patient [2]. To our knowledge, this is the first report of an endoscopic drainage of an ITF abscess through the maxillary sinus. This pathway has been described previously for resection of tumors of the ITF such as papillomas and schwannomas [1]. Similarly, endoscopic sinus surgery has been used routinely to drain periorbital abscesses as in cases of complicated sinusitis. 

## 4. Conclusion

We present a case of ITF abscess involving the pterygoid muscles following dental extraction for an odontogenic infection. This case report describes the use of nasal endoscopic sinus surgery as a tool in the management of deep space infections such as the ITF.
